# The pivotal role of endoplasmic reticulum in FDG uptake in cancer cells

**DOI:** 10.1186/s13550-024-01124-3

**Published:** 2024-07-12

**Authors:** Francesca Vitale, Maddalena Ghelardoni, Sabrina Chiesa, Sonia Carta, Serena Losacco, Anna Maria Orengo, Silvia Bruno, Silvia Ravera, Matteo Bauckneht, Mattia Riondato, Isabella Donegani, Edoardo Dighero, Jonathan Martinelli, Cecilia Marini, Gianmario Sambuceti

**Affiliations:** 1 Nuclear Medicine Unit, IRCCS Ospedale Policlinico San Martino, Largo Rosanna Benzi 10, 16133 Genoa, Italy; 2 Department of Experimental Medicine, Human Anatomy, University of Genoa, Genoa, Italy; 3 Nuclear Medicine, Department of Health Science, University of Genoa, Genoa, Italy; 4 CNR Institute of Bioimages and Molecular Physiology, Milan, Italy

## Introduction

The rapid growth of PET imaging in oncology largely reflects the user-friendly kinetic features of 2-[^18^F]-fluoro-2-deoxy-D-glucose (FDG). Sharing with glucose both GLUT-facilitated transmembrane transport and hexokinases-catalyzed trapping phosphorylation, FDG-6P is thought to accumulate within the cytosol as a false-substrate for the enzymes triggering glycolysis or pentose phosphate pathway (PPP) [1]. This irreversible retention thus permits to offer the informative potential of PET/CT imaging to a large number of patients exploiting the possibility to map the Warburg activation of cancer lesions across the whole body with a single “static” acquisition [2, 3].

Nevertheless, all mammalian cells are equipped with a transport system encoded by the SLC37A4 gene mainly represented by the glucose-6P transporter (G6PT) that transports phosphorylated hexoses within the endoplasmic reticulum (ER) where the hydrolysis catalyzed by the glucose-6P-phosphatase beta (G6Pase-β) [4–6] might result in a significant tracer loss. Together with the evidence that metabolic pathways confined within the ER largely contribute to a significant FDG-6P processing [7–11] in cancer, this consideration suggests that FDG uptake might reflect the competition between FDG-6P metabolism and hydrolysis within the ER rather than the glycolytic flux in the cytosol. Sato et al. reported a high FDG uptake in the liver in a patient with glycogen storage disease Ib, characterized by an impaired G6PT function [12]. However, whether this abnormality also interferes with tracer retention in cancer has not been fully elucidated. Accordingly, the present study aimed to evaluate whether G6PT silencing does affect the kinetics of tracer uptake in cancer cells.

## Materials and methods

### Cell culture, short-interfering RNA (siRNA) transfection and FDG kinetics

Murine breast cancer cells (4T1, ATCC, LGC Standards Srl, Milan, Italy) were cultured in complete RPMI (11 mM glucose). Cells were seeded to reach 60–80% confluency to be transfected for 48 h with G6PT siRNA (cat: #43-908-43) or mock siRNA (cat: #43–908-43) according to manufacturer instructions (Invitrogen, Waltham, USA, cat: #13,778,075).

Silenced and control cells (400,000) were cultured as a spot for 24 h. For the experiment, cultures were exposed to media with either 0 or 5.5 mM glucose and 1.8–2.2 MBq/mL FDG whose uptake was evaluated using LigandTracer White^®^ (Ridgeview, Uppsala SE) according to our standard procedure [13, 14]. After 120 min, the culture was exposed to an FDG-free medium containing the same glucose concentration to evaluate the washout rate.

### RT-PCR and metabolic analysis

Gene expression was evaluated by real-time polymerase chain reaction (RT-PCR) as previously described using the Realplex Software (Eppendorf) [15].

G6PT and G6Pase-β primers sequences:

G6PT: SLC37A4-F: TTCAACCGCAAAACCTTCTC, and SLC37A4-R: AAACTTGCTGATGGCGTAGG.

G6Pase-β: G6PC3-F: CCGGGCTAGAGAATATGTGG, and G6PC3-R: AAAAGGGCCTGTCTCCAAAC.

m-TBP was employed as housekeeping gene.

To estimate glycolysis rate, we exploited the Seahorse-XFp Extracellular Flux Analyzer (Agilent Technologies, USA) and the Glycolysis Stress Test Kit according to manufacturer instructions. In brief, this approach measures the proton efflux rate (PER). This index represents the rate of lactate release and was thus doubled to estimate the glucose consumption in picomol × min^−1^ × million cells^−1^.

### Confocal microscopy

Access of phosphorylated hexoses to the ER was evaluated by confocal microscopy (Leica Microsystems, Germany) using the FDG analogue 2-[N-(7-nitrobenz-2-oxa-1,3-diazol-4-yl)amino]-2-deoxyglucose (2-NBDG, 50 µM) and the ER tracker glibenclamide (0.5 µM) as previously described [13, 14]. A minimum of four randomly selected fields were analyzed in three independent samples for each treatment using the Fiji software (Version 2.3, NIH) to estimate colocalization according to Costes method.

### Statistical analysis

All experiments were done at least in triplicate. Data are presented as mean ± standard deviation (SD) and compared using Student’s t test. A probability value *p* < 0.05 was considered significant.

## Results

**Fig. 1:**
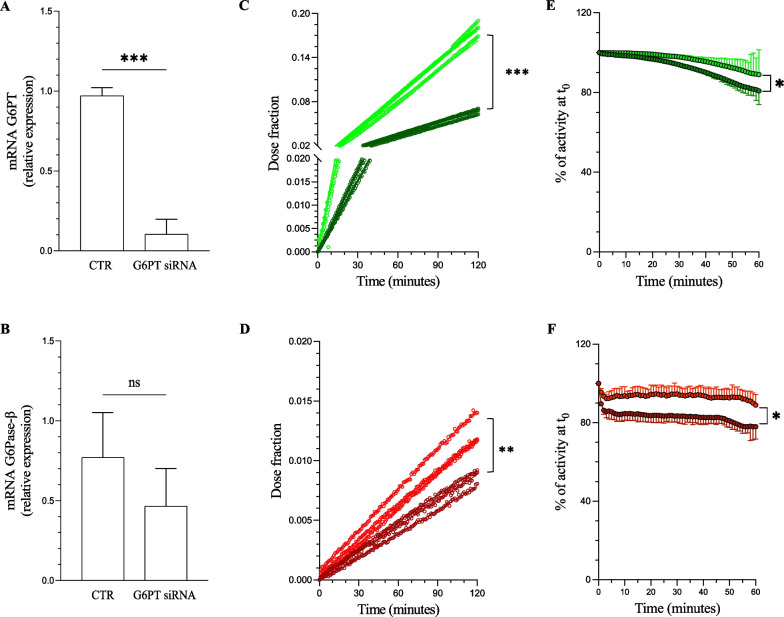
FDG uptake and washout in G6PT silenced cells. **A** SLC37A4 and **B** G6Pase-β (G6P3) mRNA expression 48 h after siRNA transfection normalized for the housekeeping gene levels. (*n* = 4, *** = *p* < 0.001). Representative FDG time-activity curves in cultures exposed to glucose-free (**C**–**E** green) or 5.5 mM glucose media (**D**–**F**, red). Silenced cells are light while their relative controls are dark. **G** and **H** FDG washout rate evaluated after switching to the cold medium (t_0_). * = *p* < 0.05, ** = *p* < 0.01, *** = *p* < 0.001 vs corresponding controls

siRNA downregulated G6PT gene expression by almost 90% (Fig. 1A), as opposed to the virtual ineffectiveness of scramble administration (data not shown). By contrast, mRNA levels of G6Pase-β were well represented and slightly (though not significantly) decreased after G6PT silencing (Fig. 1B).

Cell radioactivity content showed a progressive and virtually linear increase over the two hours observation period in both silenced and control cells (Fig. 1C-D). Expectedly, the coincubation with unlabeled glucose markedly (ninefold) decreased the radioactivity accumulation (Fig. 1C-D). By contrast, G6PT gene silencing markedly increased tracer retention by 2.5-fold in the absence of competing glucose (Fig. 1C, *p* < 0.001) and by 1.5-fold in its presence (Fig. 1D, *p* < 0.01). During the subsequent exposure to the FDG-free medium, all cultures showed a measurable washout that was, however, characterized by a lower rate in silenced than in control cells (Fig. 1E-F), regardless the presence or the absence of competition by unlabeled glucose.

**Fig. 2:**
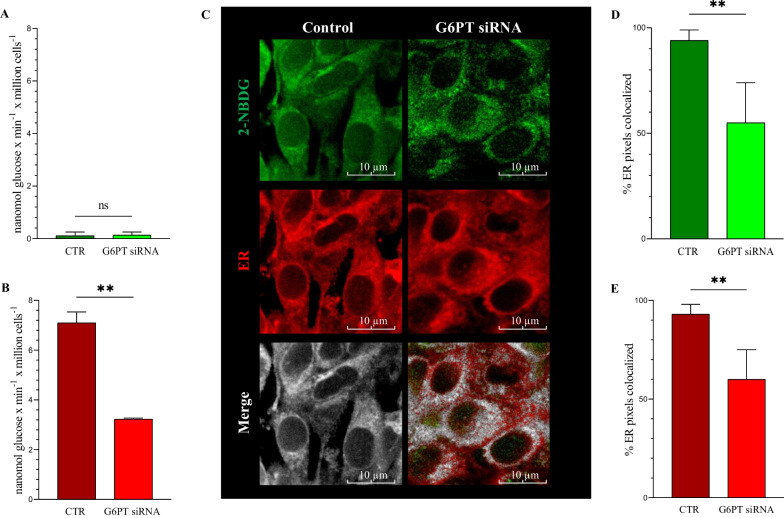
Glucose consumption and 2-NBDG uptake into the ER. Glycolytic flux was virtually absent in cells exposed to glucose free media (**A**) and almost halved by G6PT silencing in cultures exposed to a 5 mM (1 g/L) glucose concentration (**B**) (** = *p* < 0.01). Panel **C** displays representative confocal microscopy images of control and G6PT-silenced cells obtained by vital co-staining with 2-NBDG (green) and the ER tracker glibenclamide (red). Merge images show colocalization of 2-NBDG and ER staining in white. Percentage of 2-NBDG stained pixels containing glibenclamide (**D**) and percent glibenclamide stained pixels containing 2-NBDG (**E**) were lower in silenced than in control cells (** = *p* < 0.01)

However, this response was largely independent of glucose consumption. As expected, PER (and thus lactate release) was absent in glucose-deprived cultures regardless of the gene expression profile (Fig. 2A). Nevertheless, glycolytic rate was almost halved by G6PT silencing in the cultures exposed to a physiological hexose concentration (Fig. 2B).

Finally, the transport of phosphorylated hexoses across the reticular membrane was documented by the co-localization analysis of confocal microscopy images (Fig. 2C). Indeed, G6PT silencing decreased the fraction of 2-NBDG containing pixels stained by the reticular probe (Fig. 2D) as well as the fraction of glibenclamide retaining ones accumulating the fluorescent FDG analogue (Fig. 2E).

## Discussion

The present data document that the inhibition of FDG-6P transport across the reticular membrane slows down tracer release, or, in other words, that a significant fraction of FDG-6P is transported across the reticular membrane into the ER lumen. In this compartment, the accumulated radioactivity can be either released or retained according to the competition between the G6Pase-β-catalyzed hydrolysis or the access to a processing pathway able to prevent the metabolite access to the dephosphorylation.

Although somewhat disregarded, a measurable FDG washout from cancer had been already described since the introduction of the 2-deoxyglucose method to study brain metabolism [16]. A significant tracer release has been thereafter reported in cultured cells [17] and in cancer lesion evaluated both in the experimental [18, 19] and in the clinical setting [20]. The present data explain these findings, establishing a connection between radioactivity loss and FDG-6P access to the catalytic function of G6Pase-β within the ER lumen.

The evident slowdown in radioactivity washout induced by G6PT silencing paralleled a marked acceleration in tracer uptake. Altogether, these responses identify the reticular lumen as both the escape route and the accumulation site of FDG-vehiculated radioactivity. This model closely agrees with previous evidence by our [9–11, 15] and other groups [21] about the loose adherence of FDG uptake with glycolytic flux and its strict link with the activity of the hexose-6P-dehydrogenase (H6PD): the autosomal homologue of the best-recognized glucose-6P dehydrogenase, able to channel a large variety of phosphorylated hexoses to a specific PPP confined in the reticular lumen [22]. The role of this pathway in fueling the cancer needs for energy, reductive power and building blocks for cell proliferation has been demonstrated in several cancer types [11, 23].

The evident role of FDG-6P transport across the reticular membrane in modulating radioactivity retention challenges the commonly accepted models for interpreting of FDG imaging [16, 24]. On the theoretical ground, it extends to cancer cells the notion that assuming an irreversible radioactivity accumulation and thus neglecting k4 leads to an underestimation of glucose consumption, as already demonstrated in the brain [25]. By contrast, it does not affect the direct measurement of the other rate constants (k1 to k3) when their direct measurement is performed considering tracer uptake as a reversible process [26]. It also retains a potentially relevant impact on the clinical protocols. Indeed, the rate of competition between ER-PPP and G6Pase-β may vary considerably among different tumors or among different cell types populating the tumor. This variability might profoundly affect tracer retention and its distribution over time. As a matter of fact, imaging can often be delayed for various reasons after tracer injection. Current guidelines for FDG imaging require strict adherence to the minimum distribution time while the maximum interval can be prolonged up to 2 hours [2, 3]. Accordingly, the time elapsing between injection and imaging should be carefully considered, particularly when semi-quantitative indices of FDG uptake are estimated to evaluate cancer progression or response to therapy.

## Data Availability

Please contact the corresponding author.

## Abbreviations

PET/CT: Positron emission tomography/computed tomography
FDG: [^18^F]-Fluorodeoxyglucose
FDG-6P: [^18^F]-Fluorodeoxyglucose-6-phosphate
PPP: Pentose phosphate pathway
G6PT: Glucose-6-phosphate transporter
ER: Endoplasmic reticulum
G6Pase-β: Glucose-6-phosphatase-β
siRNA: Short interfering ribonucleic acid
RT-PCR: Real-time polymerase chain reaction
PER: Proton efflux rate
2-NBDG: 2-[N-(7-nitrobenz-2-oxa-1,3-diazol-4-yl)amino]-2-deoxyglucose
H6PD: Hexose-6-phosphate dehydrogenase
